# Metabolite profiling of the fermentation process of "yamahai-ginjo-shikomi" Japanese sake

**DOI:** 10.1371/journal.pone.0190040

**Published:** 2018-01-03

**Authors:** Yohei Tatsukami, Hironobu Morisaka, Shunsuke Aburaya, Wataru Aoki, Chihiro Kohsaka, Masafumi Tani, Kiyoo Hirooka, Yoshihiro Yamamoto, Atsushi Kitaoka, Hisashi Fujiwara, Yoshinori Wakai, Mitsuyoshi Ueda

**Affiliations:** 1 Division of Applied Life Sciences, Graduate School of Agriculture, Kyoto University, Sakyo-ku, Kyoto, Japan; 2 Japan Society for the Promotion of Science, Sakyo-ku, Kyoto, Japan; 3 Kyoto Industrial Science & Technology Innovation Center, Shimogyo-ku, Kyoto, Japan; 4 Kyoto Municipal Institute of Industrial Technology and Culture, Shimogyo-ku, Kyoto, Japan; 5 Advanced Science, Technology & Management Research Institute of KYOTO, Shimogyo-ku, Kyoto, Japan; 6 Kizakura Co., Ltd. Fushimi-ku, Kyoto, Japan; The University of Tokyo, JAPAN

## Abstract

Sake is a traditional Japanese alcoholic beverage prepared by multiple parallel fermentation of rice. The fermentation process of “yamahai-ginjo-shikomi” sake is mainly performed by three microbes, *Aspergillus oryzae*, *Saccharomyces cerevisiae*, and *Lactobacilli*; the levels of various metabolites fluctuate during the fermentation of sake. For evaluation of the fermentation process, we monitored the concentration of moderate-sized molecules (m/z: 200–1000) dynamically changed during the fermentation process of “yamahai-ginjo-shikomi” Japanese sake. This analysis revealed that six compounds were the main factors with characteristic differences in the fermentation process. Among the six compounds, four were leucine- or isoleucine-containing peptides and the remaining two were predicted to be small molecules. Quantification of these compounds revealed that their quantities changed during the month of fermentation process. Our metabolomic approach revealed the dynamic changes observed in moderate-sized molecules during the fermentation process of sake, and the factors found in this analysis will be candidate molecules that indicate the progress of “yamahai-ginjo-shikomi” sake fermentation.

## Introduction

Sake is a traditional Japanese rice wine prepared by multistep fermentation of rice, also called multiple parallel fermentation, in which conversions of rice starch to sugar by *Aspergillus oryzae* and sugar to alcohol by sake yeast (*Saccharomyces cerevisiae*) spontaneously occur[1]. The process of brewing sake begins with polishing of rice grains to remove bran-containing proteins, lipids, and minerals that could impair the aroma and flavor of sake. The sake using rice polished to at least 60% of the original grain was called as “ginjo” sake. Generally, a fermentation starter is prepared using steamed rice, malted rice, and sake yeast. Several types were used as ‘starter’ of the fermentation process, mainly classified to *sokujo-*type starter and yamahai*-*type (or more original type; kimoto-type) starter. For *sokujo-*type starter, yeast and lactic acid are simultaneously added to avoid the growth of contaminant bacteria. This process typically takes 1–2 weeks. In contrast, for yamahai-type starter, air-bone or certain *Lactobacilli* and yeast are added, and the inhibition of contaminant bacterial growth depends on lactic acid naturally produced by *Lactobacilli*. This process requires careful management of the fermentation process by a master brewer, and usually takes approximately 1 month, which is 2–4 times greater than the time used for sokujo*-*type. After the preparation of fermentation starter, a process called “*tome-shikomi”* (commencement of fermentation) are performed, in which fermentation starter and steamed rice are combined to yield the sake mash, and further fermented for a month. Finally, refined sake is obtained by filtration of sake mash and then bottled with or without pasteurization. “Yamahai-shikomi” sake using yamahai-type starter needs a more complex and long-term fermentation process than “sokujo-shikomi” sake, and management of the fermentation process requires fully-experienced master brewer.

Japanese sake is a target of metabolomic investigation as it usually contains many metabolites derived from both the primary rice substrate and microbial activity which are produced during parallel multiple fermentation to establish an easier quality control system instead of master brewers. There are many reports that investigated the compounds found in sake and their relationship to its sensory properties [2–5]. These studies targeted the small molecules and volatile metabolites such as amino acids, sugars, organic acids, and so on, using the capillary electrophoresis time-of-flight mass spectrometry (CE-TOFMS) or gas chromatography coupled with mass spectrometry (GC-MS). There are also a few reports that found characteristic peptides in sake [6, 7] and that targeted time-course variations of some unique peptides [8, 9]. However, there are no comprehensive reports that have focused on moderate mass compounds such as peptides and secondary metabolites during the progress of sake fermentation.

In the present study, we performed time-course metabolic profiling of “yamahai-ginjo-shikomi” sake to identify novel metabolites which characterize the progress of fermentation process. We used the nanoLC-MS system equipped with a meter-long monolithic silica capillary column and an orbitrap mass spectrometer, which is capable of accurate mass determination. We targeted molecules with mass-to-charge ratios of 200–1000 to identify the moderate-sized ones. Our research identified certain leucine- or isoleucine-rich peptides, which are the candidate molecules which characterize the fermentation process. Demonstration of brewing “yamahai-ginjo-shikomi” sake with metabolic analysis will contribute to comprehensive understanding of Japanese sake.

## Materials and methods

### Preparation of sake samples

In this study, we prepared two sake samples, sake_1 and sake_2. For the preparation of sake_1 sample, 250 kg of “Yamada Nishiki” rice harvested at Hyogo Prefecture was used with a polishing ratio of 35%. Starter of “yamahai-ginjo-shikomi” was prepared using 21.2% aliquot of rice. For preparation of starter, the malted rice was kept under around 6°C for 5 days. The temperature of starter was then gradually increased to the room temperature for 10 days. Then yeast was added and grew for 27^th^ day from starter preparation started. In total, 339 L of sake containing 15.9% of ethanol was produced, and samples were collected at 6, 11, 16, 21, 26, and 34 days after “*tome-shikomi*”.

For the preparation of sake_2 sample, 200 kg of “Yamada Nishiki” rice harvested at Tottori Prefecture was used with a polishing ratio of 35%. Starter was prepared using 20.5% aliquot of rice by the same way described above. For efficient saccharification of rice starch, 10 g of glucoamylase “Amano” SD (Amamo Enzyme Inc., Nagoya, Japan) was added at an early stage of fermentation. In total, 270 L of sake containing 16.3% of ethanol was produced, and samples were taken at 1, 5, 11, 19, 26, and 34 days after “*tome-shikomi*”. All fermentation processes of sake mash were performed under conditions of 5.8–10.4°C for both kinds of sake.

Microbial strains used for fermentation were *Leuconostoc mesenteroides* and *Lactobacillus plantarum* for starter Lactobacillales, *Saccharomyces cerevisiae* for sake yeast, and *Aspergillus oryzae* for seed malted rice. Parameters of fermentation process are shown in S1 Table.

### nanoLC-MS analysis

Non-targeted metabolomic analysis was performed using a nanoLC (Ultimate 3000, DIONEX, Sunnyvale, CA, USA)-MS system (LTQ Orbitrap Velos, Thermo Fisher Scientific). Five μl volumes of samples were injected and separated by reversed-phase chromatography using a monolithic column (2,000 mm, 0.1 mm ID, Kyoto Monotech Co., Ltd., Kyoto) [10]. The gradient was obtained by changing the mixing ratio of two eluents: A, 0.1% (v/v) formic acid and B, 80% (v/v) acetonitrile containing 0.1% (v/v) formic acid. The 120-min gradient separation at a flow rate of 500 nL/min and 40°C was as follows: (i) 5% B for 10 min, (ii) a 60-min gradient to 95% B, (iii) a 10-min hold at 95% B, (iv) a 1-min gradient return to 5% B, and (v) a 40-min hold at 5% B. The separated metabolites were detected by mass spectrometry using a full scan range of 200–1000 m/z at a resolution of 100,000. MS was performed with an ESI voltage of 2.3 kV and a transfer tube temperature of 280°C. MS data acquisition was set at 30–115 min.

For data-dependent MS/MS spectrum acquisition, which was used for peptide identification, the method was set to automatically analyze the top 10 most intense ions observed during the MS scan. Target peptides were synthesized using Genscript (Piscataway, NJ, USA).

### Data analysis

Data were acquired using the following procedures: 1) raw data obtained by LTQ Orbitrap Velos (which comprised 3,000 spectrum counts and 800 Th of mass-to-charge ratio) were delimited by 50 spectrum counts and a mass-to-charge ratio of 1, 2) the intensities in each compartment were totaled, and 3) data were exported in text file format by an in-house developed C-script named OrbitrapDataRead (Raw data and OrbitrapDataRead are available at http://www.ebi.ac.uk/metabolights/MTBLS451). The data obtained from sake samples were analyzed and integrated into the same CSV file. In this step, all ion signals are included in numeric information of CSV file without discrimination of noise signals from metabolite-related signals. The detailed workflow of OrbitrapDataRead was described in S1 Fig. Global median normalization was conducted to standardize the total intensities of compounds injected into the LC-MS. Finally, principal component analysis (PCA) was performed using R (https://www.r-project.org/).

### Quantification of compounds

Quantification of the two peptides of interest (see in Results) was performed by LC-triple quadrupole mass spectrometry (LC-TQMS; LC-MS-8060, Nexela system, Shimadzu, Kyoto, Japan). Samples were injected and separated by reversed-phase chromatography using InertSustain AQ-C18 (150 mm, 2.1 mm ID, 2.0 μm particle size, GL Science, Osaka, Japan) at a flow rate of 400 μL/min. The gradient was obtained by changing the mixing ratio of two eluents: A, 0.1% (v/v) formic acid and B, acetonitrile containing 0.1% (v/v) formic acid. The 23 min gradient separation at a flow rate of 400 μL/min and 40°C was as follows: (i) 5% B for 2 min, (ii) a 15-min gradient to 45% B, (iii) an immediate increase to 95% B, (iv) a 3 min hold at 95% B, (v) an immediate decrease back to 5% B, and (vi) a 3-min hold at 5% B. The parameters of Multiple Reaction Monitoring were set as follows: (i) polarity, positive; transition, 358.15 > 86.05; Q1 Pre-bias: −20 V; collision energy (CE), −30 V; Q3 Pre-bias: −20 V for Leu–Leu–Leu and (ii) polarity, positive; transition, 376.20 > 229.20; Q1 Pre-bias: −20 V; collision energy (CE), −20 V; Q3 Pre-bias: −20 V for Phe–Pro–Leu.

Relative quantification of C_19_H_28_O, C_17_H_26_N_2_O_3_, [Leu/Ile]–[Leu/Ile]–[Leu/Ile]–Pro, and [Leu/Ile]–[Leu/Ile]–[Leu/Ile]–[Leu/Ile]–Pro was performed using extracted ion chromatograms of metabolome profiles obtained by nanoLC-MS.

## Results

### Metabolomics of “yamahai-ginjo-shikomi” sake during fermentation process

**Fig 1:**
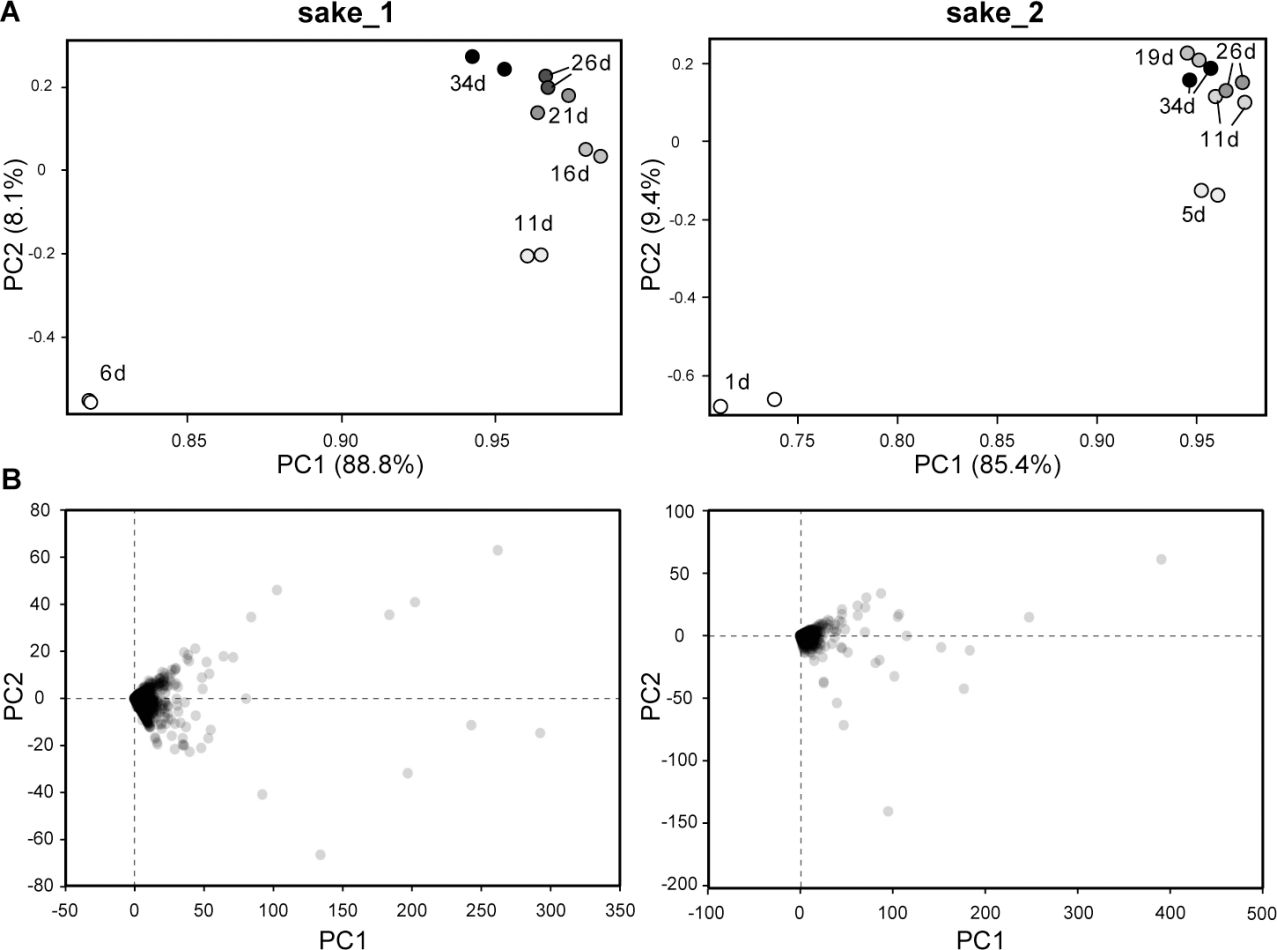
PCA analysis of metabolic profiles. (A) Score plot of the fermentation process. Metabolic profiles of samples at six time points were plotted using two analytical replicates from the same sample. (B) Loading scatter plot. Factors having values of PC1 from the top to the 20^th^ at each plot are shown in Table 1.

We used examples of sake mash from two samples identified as sake_1 and sake_2. To profile the metabolite changes of the fermentation processes, we prepared the sake mash samples taken during sake fermentation process at 6, 11, 16, 21, 26, and 34 days after “*tome-shikomi*” for sake_1 sake mash sample, and at 1, 5, 11, 19, 26, and 34 days after “*tome-shikomi*” for sake_2 sake mash samples. Supernatants of the prepared samples were then analyzed using LC-MS. PCA of the acquired data showed that the spectral profiles of the sake mash changed in a phased manner during fermentation, as well as exhibited high levels of similarity between the two technical replicates (Fig 1A). The PCA loading scatter plot exhibited certain features that profile the progress of fermentation process (Fig 1B). Factors having values of principal component 1 (PC1) from the top to the 20th were extracted (Table 1). Then the common factors in both sake_1 and sake_2 were focused (described in **underlined bold** numbers in Table 1). For detailed analysis, the main exact mass occupying each factor was extracted from spectral data. Each exact mass of ≤1000 Da was analyzed using the Kazusa Molecular Formula Searcher (http://webs2.kazusa.or.jp/mfsearcher/index_jp.html) with KEGG (http://www.genome.jp/kegg/), KNApSAcK (http://kanaya.naist.jp/KNApSAcK/) and pep1000 (http://webs2.kazusa.or.jp/mfsearcher/pep1000/) databases to predict candidate chemicals or peptides (Table 2). Exact masses of ≥ 1000 Da could not be determined because of the redundancy of molecular formula. From the results of compound predictions, C_17–29_ organic compounds, including leucine- or isoleucine-rich short-chain peptides, exhibited the highest PC1 scores.

**Table 1:** PC scores with Top 20 PC1 score.

Sake_1					Sake_2				
No.	m/z	# Spectra	PC1	PC2	No.	m/z	# Spectra	PC1	PC2
**1**	752–753	1400–1450	291.1	-14.6	**1**	455–456	1250–1300	390.2	60.7
**2**	455–456	1250–1300	260.4	62.8	**2**	754–755	1350–1400	247.3	14.7
**3**	751–752	1400–1450	241.6	-11.2	**3**	752–753	1400–1450	183.2	-11.6
**4**	273–274	1350–1400	201.2	40.9	**4**	391–392	2600–2650	176.7	-42.4
**5**	376–377	1200–1250	195.8	-31.7	**5**	751–752	1400–1450	152.1	-9.4
**6**	754–755	1350–1400	182.7	35.5	**6**	273–274	1350–1400	115.0	-0.2
**7**	391–392	2600–2650	133.3	-66.1	**7**	456–457	1250–1300	106.8	17.0
8	307–308	1400–1450	102.1	46.1	8	568–569	1400–1450	105.1	15.0
**9**	803–804	2600–2650	91.4	-40.6	**9**	376–377	1200–1250	101.6	-32.7
**10**	568–569	1400–1450	83.4	34.5	10	391–392	2550–2600	94.8	-140.2
**11**	688–689	1400–1450	79.9	0.0	11	832–833	1200–1250	87.2	33.7
**12**	456–457	1250–1300	70.4	17.4	**12**	803–804	2600–2650	85.6	-19.5
13	404–405	1450–1500	63.6	17.9	**13**	662–663	1350–1400	81.4	-21.9
**14**	391–392	2650–2700	54.5	-13.2	14	483–484	1550–1600	71.4	30.4
**15**	755–756	1350–1400	53.4	10.5	15	307–308	1400–1450	70.2	22.4
16	605–606	1000–1050	52.8	-16.8	**16**	755–756	1350–1400	69.8	2.8
**17**	358–359	1200–1250	51.4	15.4	**17**	358–359	1200–1250	62.0	15.9
**18**	662–663	1350–1400	48.7	4.2	18	830–831	1250–1300	61.7	23.6
19	216–217	1950–2000	48.2	8.9	19	338–339	2700–2750	51.3	-13.2
20	804–805	2600–2650	47.6	-20.9	**20**	391–392	2650–2700	48.6	5.0

Components with **underlined bold** characters indicate common factors shared between the two batches of sake

**Table 2:** Characteristic compounds.

ID	Factor		Spectrum information			Calculated exact mass	Molecular formula	Mw	Candidate compound
	m/z	# Spectra	T_*R*_ (min)	exact m/z	Charge				
1	273–274	1350–1400	68.8	273.217	+1	272.210	C_19_H_28_O	272.2140	Androstenone
2	307–308	1400–1450	70.0	307.201	+1	306.194	C_17_H_26_N_2_O_3_	306.1962	Angeloyloxylupanine, Acetoxymatrine
3	358–359	1200–1250	64.9	358.271	+1	357.264	C_18_H_35_N_3_O_4_	357.2628	[L/I]_3_
4	376–377	1200–1250	64.0	376.223	+1	375.216	C_20_H_29_N_3_O_4_	375.2158	[L/I]FP
5	391–392	2600–2700	102–105	391.284	+1	390.277	C_24_H_38_O_4_	390.2770	Diisooctyl phthalate [M + H]^+^
6	455–456	1250–1300	65.9	445.322	+1	454.315	C_23_H_42_N_4_O_5_	454.3155	[L/I]_3_P
	456–457	1250–1300	Identical molecule of ID 6 [m/z: 455–456, # Spectra: 1250–1300] with one isotopic atom						
7	568–569	1400–1450	70.1	568.406	+1	567.399	C_29_H_53_N_5_O_6_	567.3996	[L/I]_4_P
8	662–663	1350–1400	69.2	662.309	+2	1322.604	N.A.		
9	751–752	1400–1450	70.1	751.863	+2	1501.712	N.A.		
	752–753	1400–1450	Identical molecule of ID 9 [m/z: 751–752, # Spectra: 1400–1450] with one isotopic atom						
10	754–755	1350–1400	68.9	754.351	+2	1506.688	N.A.		
	755–756	1350–1400	Identical molecule of ID 10 [m/z: 754–755, # Spectra: 1350–1400] with two isotopic atoms						
11	803–804	2600–2650	102–104	803.542	+1	802.535	C_48_H_76_O_8_Na	803.5432	Diisooctyl phthalate [M_2_ + Na]^+^

Factors shared between sake_1 and sake_2 (represented in **underlined bold** characters in Table 1) are shown. [L/I] indicates leucine or isoleucine residues that could not be determined by MS. N.A.; Not annotated. The factor ID 5 (m/z: 391–392. # spectra: 2600–2700) and ID 11 (m/z: 804–805, # spectra: 2600–2650) were identified as diisooctyl phthalate (di(2-ethylhexyl)phthalate; DHEP), which is a major plasticizer used in plastic flow channel of LC. DHEP is inevitably detected in MS and become analytical background.

### Identification of characteristic peptides

**Fig 2:**
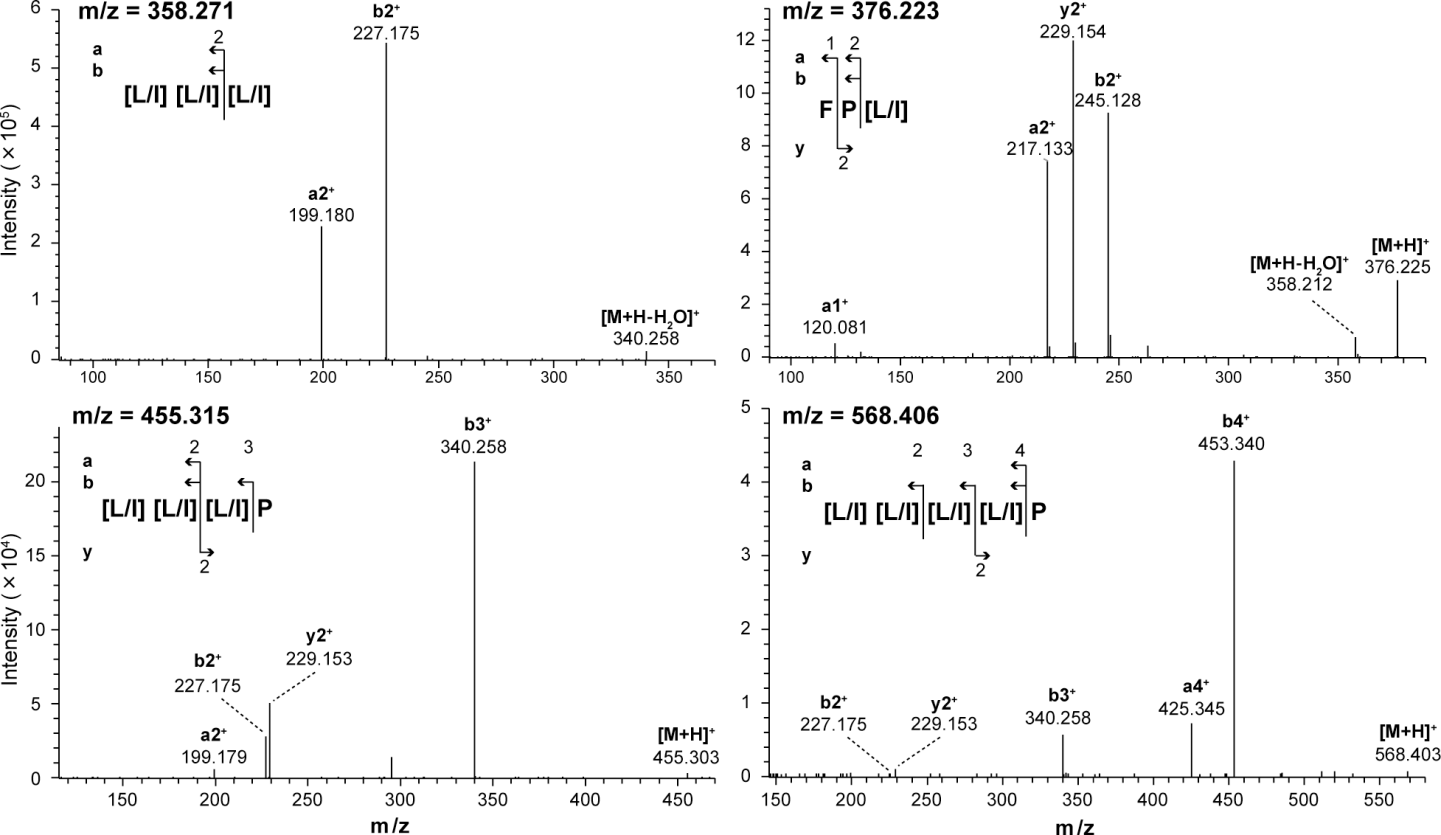
Identification of peptides. The factors that were predicted as peptides in Table 2 were analyzed using LC-MS/MS, and the amino acid sequences were identified using MS/MS spectra. [L/I] indicates leucine or isoleucine residue that could not be distinguished by MS analysis.

To identify candidate compounds which can characterize the progress of fermentation process, we performed nanoLC-MS/MS analysis. We particularly focused on the leucine- or isoleucine-rich peptides, and the sequences of these peptides were analyzed. As a result, the peptides [Leu/Ile]–[Leu/Ile]–[Leu/Ile], Phe–Pro–[Leu/Ile], [Leu/Ile]–[Leu/Ile]–[Leu/Ile]–Pro, and [Leu/Ile]–[Leu/Ile]–[Leu/Ile]–[Leu/Ile]–Pro were identified (Fig 2). All peptides contained leucine or isoleucine (represented as [Leu/Ile]). However, leucine and isoleucine could not be distinguished by MS analysis because these two amino acid residues share the same molecular weight.

**Fig 3:**
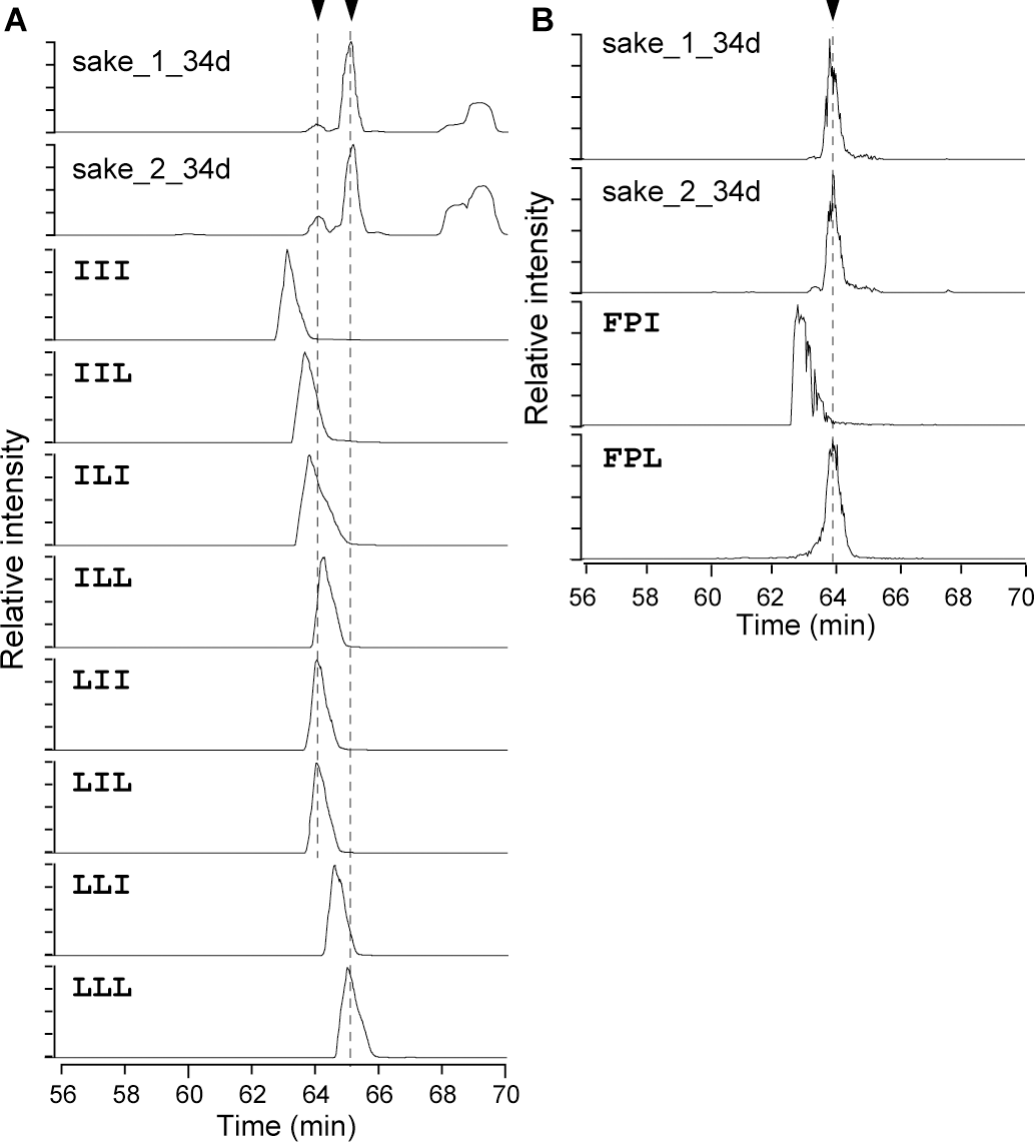
Validation of peptide leucine or isoleucine compositions. (A) Chromatograms of sake samples with eight variations of synthetic [Leu/Ile]–[Leu/Ile]–[Leu/Ile] peptides. Extracted ion chromatograms (XIC) of m/z = 358.26–358.28 are presented. (B) Chromatograms of sake samples with two variations of synthetic Phe–Pro–[Leu/Ile] peptides. XIC of m/z = 376.22–376.23 are shown. Dashed lines with arrowheads indicate retention times of the main peaks of XIC for two samples. [Leu/Ile] indicates leucine or isoleucine residues that could not be determined by MS.

Next, we tried to distinguish leucine and isoleucine residues by their retention time on chromatographic columns using (as an example) the tripeptides [Leu/Ile]–[Leu/Ile]–[Leu/Ile], which display 2^3^ patterns of leucine and/or isoleucine sequence. The long-duration gradient program employed for LC using the long monolithic column was able to distinguish these tripeptides (Fig 3A). From the chromatogram, the sample contained more than one species of peptide, and the predominant one was found to be Leu–Leu–Leu, indicating that peptides possessing a poly-leucine motif may be important. Moreover, putative Phe–Pro–[Leu/Ile] was validated as Phe–Pro–Leu by the retention time analysis using LC (Fig 3B), suggesting that leucine-containing peptides are the compounds that showed highest PC1 scores in our samples. The precise composition of leucine and isoleucine peptides for [Leu/Ile]–[Leu/Ile]–[Leu/Ile]–Pro and [Leu/Ile]–[Leu/Ile]–[Leu/Ile]–[Leu/Ile]–Pro could not be identified because of the number of sequence combinations.

### Quantification of fermentation-characteristic peptides

**Fig 4:**
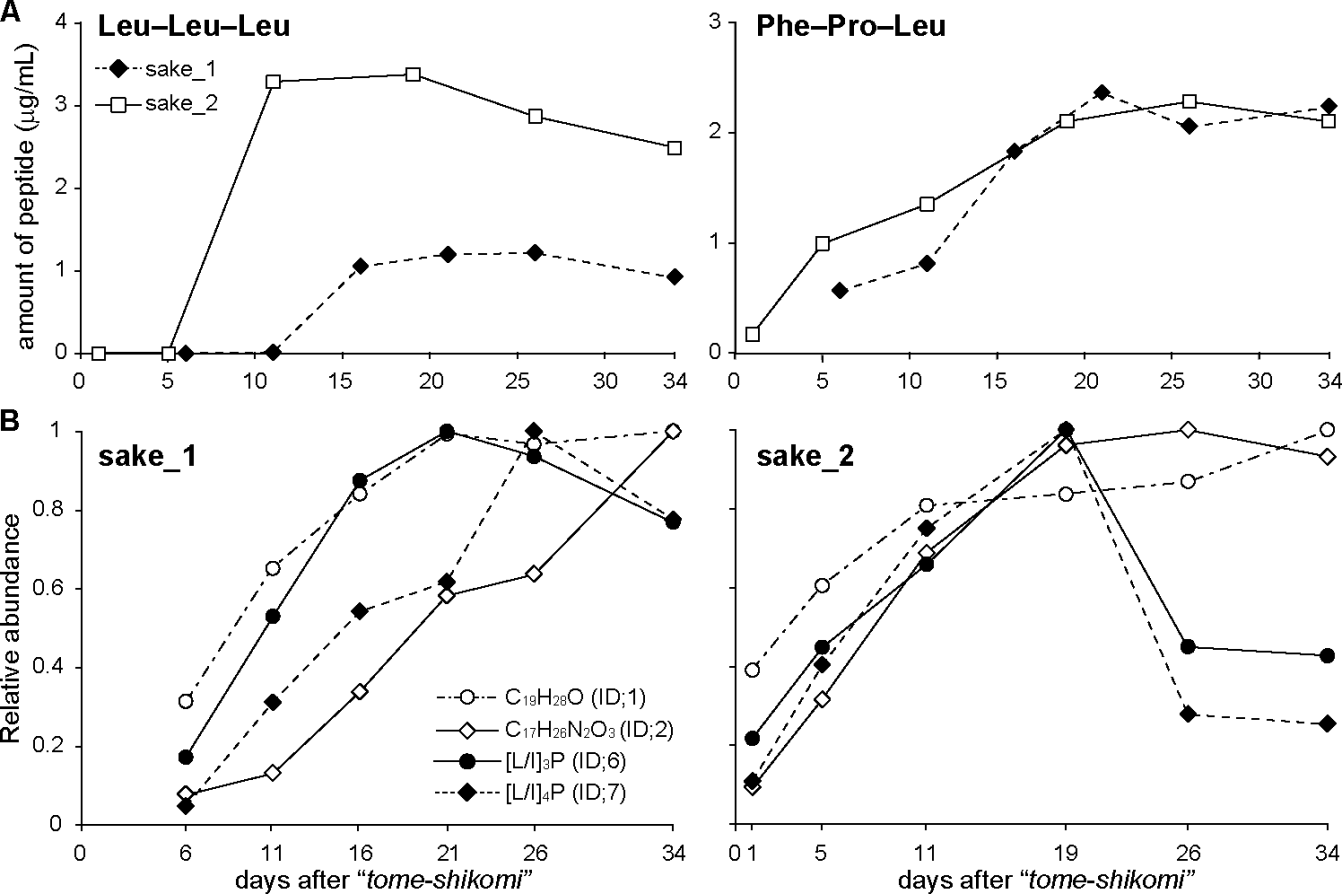
Quantification of peptides and associated compounds. (A) Quantification of Leu–Leu–Leu (left) and Phe–Pro–Leu by LC-TQMS. (B) Relative quantification of four factors (ID 1, 2, 6, and 7) that were identified by metabolic profiling using nanoLC-MS.

To confirm quantitative changes involving the peptides found by metabolic profiling using our LC-MS analytical system, polypeptides were quantified by LC-triple quadrupole (TQ) MS. Because eight variants of [Leu/Ile]–[Leu/Ile]–[Leu/Ile] cannot be separated by the LC-TQMS system, the quantity of peptides was determined using a standard calibration curve of Leu–Leu–Leu, which was the main component of the peptides (Fig 3A). From the results of quantification (Fig 4A), it was observed that the levels of Leu–Leu–Leu and Phe–Pro–Leu dramatically increased to the level of micrograms per milliliter during the early stages of fermentation process. Relative abundances of C_19_H_28_O, C_17_H_26_N_2_O_3_, [Leu/Ile]–[Leu/Ile]–[Leu/Ile]–Pro, and [Leu/Ile]–[Leu/Ile]–[Leu/Ile]–[Leu/Ile]–Pro were also elevated during the fermentation process of the sample (Fig 4B).

## Discussion

In this study, we performed the time-course metabolic profiling of “yamahai-ginjo-shikomi” Japanese sake and first identified factors that indicate the progress of the fermentation process. By targeting moderate-sized molecules with mass-to-charge ratios of 200–1000, signature biomolecules produced during the fermentation process were revealed by PCA (Fig 1A). Analysis indicates that the profiles of these moderate-sized molecules changed during fermentation. Moreover, the metabolite profile of moderate-sized molecules changed more dramatically during the early fermentation stages (<11 days) than in later stages (≥11 days), indicating that early fermentation stages determine the metabolic profile of sake.

Four peptides which contain leucine or isoleucine were identified by metabolic profiling (Fig 2). From the separation of [Leu/Ile]–[Leu/Ile]–[Leu/Ile] (Fig 3A), the dominant peptide was found to be Leu–Leu–Leu, suggesting that the peptides identified in this study share leucine-rich motifs. Therefore, the other two peptides were suggested to be Leu–Leu–Leu–Pro and Leu–Leu–Leu–Leu–Pro. These three peptides may be derived from the same protein precursor. Leucine is a hydrophobic amino acid, and leucine-rich motifs are typically associated with transmembrane α-helical structures [11] and membrane-anchor domains of proteins [12]. The leucine-rich motif, including Leu–Leu–Leu–Leu–Pro is more abundant in proteins encoded in the genome of *Oryza sativa* (rice) than those of *S*. *cerevisiae* (sake yeast), *A*. *oryzae*, or *Lactobacilli* as revealed by BLAST homology identification, suggesting that the peptides found in this study could be generated during digestion of rice proteins by proteases of inoculated yeast, *Lactobacilli*, *A*. *oryzae*, or externally added glucoamylase which is crude enzyme mixture and has peptidase and protease activity.

The peptides revealed in this study possibly affect the taste of sake. Branched chain amino acids such as valine, leucine, and isoleucine are generally considered to confer bitter taste sensations [13]. Leucine-containing peptides are also known to confer bitterness [14–16], but the bitterness thresholds of these peptides are the milligram per milliliter level, and the concentration of the peptides in our sample was microgram per litter level. There is another report that indicates Leu–Leu–Leu has a taste-masking effect of sourness [17], indicating that the peptides identified in this study may affect the taste of “yamahai-ginjo-shikomi” sake. Our results indicated that the amount of Leu–Leu–Leu and Phe–Pro–Leu were elevated up to the order of μg/mL, indicating that these peptides likely characterize the taste of sake.

In this study, two factors other than peptides were also identified by metabolic profiling (Table 2). ID 1 (m/z: 273–274, # spectra: 1350–1400), identified as C_19_H_28_O, was categorized as a likely steroidal agent, including androstenone. Androstenone, a well-known mammalian sex hormone, can also be detected in plants [18], and is reported to be resemble a sweaty, urine or woody smell, or even a pleasant floral odor, this perceptual variation most likely depends on the olfactory receptor variant genotypes of individuals subjected to exposure [19]. The factor ID 2 (m/z: 307–308, # spectra: 1400–1450) identified as C_17_H_26_N_2_O_3_ was predicted to be angeloyloxylupanine or acetoxymatrine. Angeloyloxylupanine and acetoxymatrine are plant alkaloids [20, 21], which generally exert bitter taste sensations, and they are predicted to appear during rice seed decomposition by yeast or *A*. *oryzae*. These factors require further researches with authentic standards.

Here, we first demonstrated time-course metabolic profiling targeting moderate-sized molecules during fermentation of “yamahai-ginjo-shikomi” sake. Metabolic profiling revealed that the abundance profiles of these moderate-sized molecules were dependent on the specific fermentation stage employed. Analysis of characteristic and distinguishing ions by LC-MS/MS revealed that four leucine- (or isoleucine-) containing short peptides became elevated during the fermentation process. These peptides would be likely candidate biological molecules that reveal and indicate the progress of sake fermentation, and require further investigation and validation.

## Supporting information

S1 Table Parameters of fermentation process. This material shows shikomi-haigo, alcohol concentration, glucose concentration, and nihonshudo (sake metre value) of sake_1 and sake_2
(XLSX)

S1 Fig Program map of OrbitrtapDataRead. This map represents the data processing method of OrbitrapDataRead against raw file produced by LTQ Orbitrap Velos.
(TIF)

## References

[1] Steinkraus KH. Fermentations in world food processing. Com Rev Food Sci Food Saf. 2002;1:23–32. 10.1111/j.1541-4337.2002.tb00004.x 33451246

[2] Mimura N, Isogai A, Iwashita K, Bamba T, Fukusaki E. Gas chromatography/mass spectrometry based component profiling and quality prediction for Japanese sake. J Biosci Bioeng. 2014;118(4):406–14. doi: 10.1016/j.jbiosc.2014.04.006. WOS:000345189300009. 25060729 10.1016/j.jbiosc.2014.04.006

[3] Sugimoto M, Kaneko M, Onuma H, Sakaguchi Y, Mori M, Abe S, et al Changes in the Charged Metabolite and Sugar Profiles of Pasteurized and Unpasteurized Japanese Sake with Storage. J Agr Food Chem. 2012;60(10):2586–93. doi: 10.1021/jf2048993. WOS:000301407000024. 22352923 10.1021/jf2048993

[4] Sugimoto M, Koseki T, Hirayama A, Abe S, Sano T, Tomita M, et al Correlation between Sensory Evaluation Scores of Japanese Sake and Metabolome Profiles. J Agr Food Chem. 2010;58(1):374–83. doi: 10.1021/jf903680d. WOS:000273268100051. 19961224 10.1021/jf903680d

[5] Takahashi K, Kohno H. Different Polar Metabolites and Protein Profiles between High- and Low-Quality Japanese Ginjo Sake. Plos One. 2016;11(3). ARTN e0150524 doi: 10.1371/journal.pone.0150524. WOS:000371735200100. 26939054 10.1371/journal.pone.0150524 PMC4777507

[6] Kiyono T, Hirooka K, Yamamoto Y, Kuniishi S, Ohtsuka M, Kimura S, et al Identification of pyroglutamyl peptides in Japanese rice wine (sake): presence of hepatoprotective pyroGlu-Leu. J Agric Food Chem. 2013;61(47):11660–7. doi: 10.1021/jf404381w . 24175632 10.1021/jf404381w

[7] Kiyono T, Wada S, Ohta R, Wada E, Takagi T, Naito Y, et al Identification of pyroglutamyl peptides with anti-colitic activity in Japanese rice wine, sake, by oral administration in a mouse model. J Funct Food. 2016;27.

[8] Iemura Y, Yamada T, Takahashi T, Furukawa K, Hara S, Influence of amino acid content in seed mash on peptide uptake by yeast cells in main mash in sake brewing process. J Biosci Bioeng. 1999:88(6):679–81 16232685 10.1016/s1389-1723(00)87101-7

[9] Iemura Y, Takahashi T, Yamada T, Furukawa K, Hara S, Properties of TCA-insoluble peptides in kimoto (traditional seed mash for sake brewing) and conditions for liberation of the peptides from rice protein. J Biosci Bioeng. 1999:88(5):531–35 16232657 10.1016/s1389-1723(00)87671-9

[10] Miyamoto K, Hara T, Kobayashi H, Morisaka H, Tokuda D, Horie K, et al High-Efficiency Liquid Chromatographic Separation Utilizing Long Monolithic Silica Capillary Columns. Anal Chem. 2008;80(22):8741–50. doi: 10.1021/ac801042c. WOS:000260910900052. 18947204 10.1021/ac801042c

[11] Pace CN, Scholtz JM. A helix propensity scale based on experimental studies of peptides and proteins. Biophys J. 1998;75(1):422–7. WOS:000075246200040. 9649402 10.1016/s0006-3495(98)77529-0 PMC1299714

[12] Whitley P, Grahn E, Kutay U, Rapoport TA, vonHeijne G. A 12-residue-long polyleucine tail is sufficient to anchor synaptobrevin to the endoplasmic reticulum membrane. J Biol Chem. 1996;271(13):7583–6. WOS:A1996UC77400059. 8631791 10.1074/jbc.271.13.7583

[13] Mukai J, Tokuyama E, Ishizaka T, Okada S, Uchida T. Inhibitory effect of aroma on the bitterness of branched-chain amino acid solutions. Chem Pharm Bull. 2007;55(11):1581–4. doi: 10.1248/Cpb.55.1581. WOS:000251592600007. 17978515 10.1248/cpb.55.1581

[14] Gardner RJ. Correlation of Bitterness Thresholds of Amino-Acids and Peptides with Molecular Connectivity. J Sci Food Agr. 1980;31(1):23–30. doi: 10.1002/jsfa.2740310105. WOS:A1980JF51800004.

[15] Ishibashi N, Arita Y, Kanehisa H, Kouge K, Okai H, Fukui S. Studies on Flavored Peptides .1. Bitterness of Leucine-Containing Peptides. Agr Biol Chem Tokyo. 1987;51(9):2389–94. WOS:A1987K205400011.

[16] Warmke R, H.D. B. Influence of glutamic acid on the bitter taste of various compounds. Z Lebensm Unters Forsch. 1993;197(2):132–3.

[17] Kono M, Ikeuchi M, inventors; Takasago Perfumery Co. Ltd., Taste-improving peptide. 2014: EP2730176 A1

[18] Janeczko A, Skoczowski A. Mammalian sex hormones in plants. Folia Histochem Cyto. 2005;43(2):71–9. WOS:000229429100001. 16044944

[19] Keller A, Zhuang HY, Chi QY, Vosshall LB, Matsunami H. Genetic variation in a human odorant receptor alters odour perception. Nature. 2007;449(7161):468–U6. doi: 10.1038/nature06162. WOS:000249724800042. 17873857 10.1038/nature06162

[20] Xiao P, Kubo H, Komiya H, Higashiyama K, Yan YN, Li JS, et al (-)-14 beta-acetoxymatrine and (+)-14 alpha-acetoxymatrine, two new matrine-type lupin alkaloids from the leaves of Sophora tonkinensis. Chem Pharm Bull. 1999;47(3):448–50. WOS:000079315000027.

[21] Wink M, Schiebel HM, Witte L, Hartmann T. Quinolizidine Alkaloids from Plants and Their Cell-Suspension Cultures—Ester Alkaloids of Lupinus Polyphyllus. Planta Med. 1982;44(1):15–20. doi: 10.1055/s-2007-971391. WOS:A1982MZ35600002. 17402075 10.1055/s-2007-971391

