# Supplementary figures and images for: Metabolite profiling of the fermentation process of "yamahai-ginjo-shikomi" Japanese sake
Source: PLoS One. 2018 Jan 3;13(1):e0190040. doi: 10.1371/journal.pone.0190040 (PMC5752023; doi:10.1371/journal.pone.0190040)

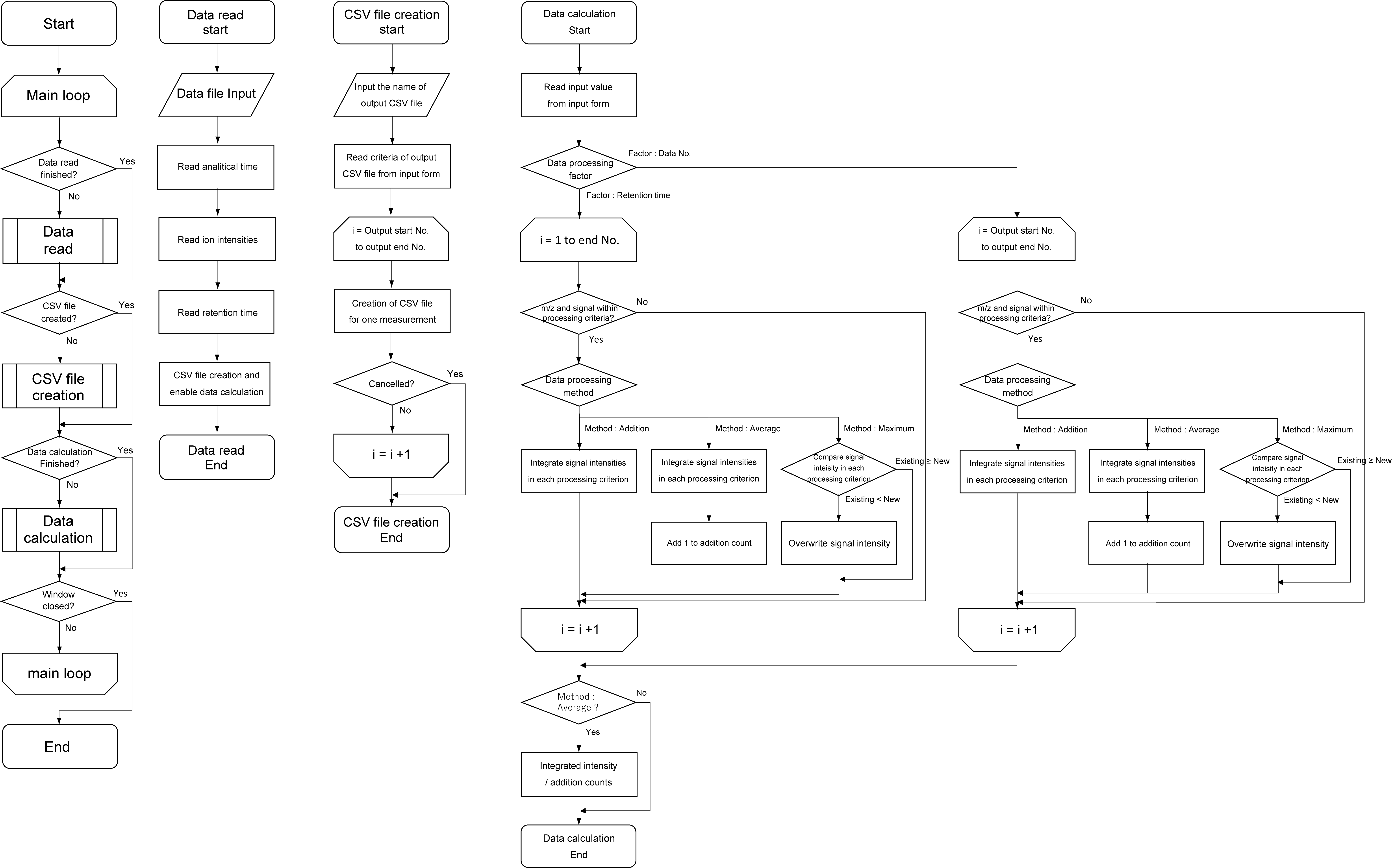

Supplement: S1 Fig — This map represents the data processing method of OrbitrapDataRead against raw file produced by LTQ Orbitrap Velos. (TIF) [file pone.0190040.s002.tif]
